# Pre-Competition Oral Findings in Danish Sport Horses and Ponies Competing at High Level

**DOI:** 10.3390/ani12050616

**Published:** 2022-03-01

**Authors:** Mette Uldahl, Louise Bundgaard, Jan Dahl, Hilary M. Clayton

**Affiliations:** 1 Vejle Equine Practice, Fasanvej 12, 7120 Vejle Øst, Denmark; mette@vejlehestepraksis.dk; 2 Louise Bundgaard, Department of Veterinary Clinical Sciences, University of Copenhagen, Agrovej 8, 2630 Taastrup, Denmark; lb@sund.ku.dk; 3 Jan Dahl Consult, Østrupvej 89, 4350 Ugerløse, Denmark; jd@lf.dk; 4 Sport Horse Science, 3145 Sandhill Road, Mason, MI 48854, USA

**Keywords:** horse, high level competition, oral pathology, oral lesions, bit use

## Abstract

**Simple Summary:**

This study addresses the presence and location of lesions in the corners of the lips, inside the cheeks, and on the bars of the mouths of 342 dressage, show jumping, and eventing horses that were examined by a veterinarian before competing in the Danish National Championships in 2020. When ulcers were present in the corners of the lips, photographs were taken for subsequent analysis. The results showed that when a cutaneous or mucosal ulcer, scar, or fissure was present at the corners of the lips on one side there was an increased risk of finding the same type of lesion on the other side. At the corners of the lips, external ulcers were correlated with internal ulcers, and both were associated with scarring and depigmentation. Erosion/contusion at the lip commissures and ulcers of the buccal mucosa were associated with similar lesions on the bars. If dental hooks or sharp enamel points were recorded on one side, they were likely bilateral. Dental findings were associated with scarring/depigmentation but not with mucosal ulcers or erosion/contusion at the lip commissures.

**Abstract:**

This study addresses the presence and location of oral lesions in 342 dressage, show jumping, and eventing horses examined at an obligatory veterinary inspection before competing in the Danish National Championship in 2020. Ulcers in the lip commissures were photographed for subsequent pathological analysis. If a lesion was found at the lip commissures on one side, there was an increased risk of finding a similar lesion on the other side (ulcer: *p* < 0.0001; scarring/depigmentation: *p* < 0.0001; fissure: *p* = 0.002; erosion/contusion: *p* < 0.0001). At the lip commissures, external (cutaneous) ulcers were correlated with internal (mucosal) ulcers (*p* < 0.0001) and with scarring/depigmentation (*p* < 0.0001). Both mucosal and cutaneous ulcers were correlated with scarring/depigmentation (*p* < 0.0001). Erosion/contusion at the lip commissures was associated with similar lesions on the bars (*p* = 0.0002), and ulcers of the buccal mucosa were associated with ulcers on the bars (*p* = 0.003). Dental hooks or sharp enamel points on one side were associated with similar lesions on the other side (*p* < 0.0001). Dental findings were not related to mucosal ulcers or erosion/contusion at the lip commissures but were associated with scarring/depigmentation (*p* = 0.01).

## 1. Introduction

The welfare of the horses used for sporting activities must be protected both during daily training and in competitions, and this implies a need for knowledge of the mechanisms of action of equipment used to control the horse’s movements and their effects on the horse. A bit or combination of bits placed in the horse’s interdental space has a long history of use by equestrians as a means of controlling speed and direction of movement. Wear patterns on the premolar teeth of prehistoric horses indicate that bits have been used for at least 6000 years [1]. Over the centuries, the shape, materials, and mechanics of bits have changed usually with the goal of improving the rider’s control. However, the way different riders use the bit varies and this may have a decisive effect on horse welfare.

In horses examined immediately after a competition, oral lesions were visible in 9.2% of 3143 Danish horses across all disciplines and levels of competition but with a higher frequency in horses competing in higher level competitions and in dressage [2]. In 208 Finnish event horses, 52% were found to have acute lesions after completing the cross-country phase. In both studies, the inner lip commissure was the most common lesion location. Evaluation of Icelandic horses at a competition [3] found mild lesions in 36% of 424 horses at the start of the competition, with the most common location being the lip commissures and buccal mucosa. Later during the competition, 77 horses were re-evaluated and showed a large increase in lesions of the bars of the mandible specifically in association with the use of a curb bit. A high frequency of severe commissural lesions has been reported in thoroughbred racehorses, most of which wear a snaffle bit [4], and it has been suggested that this may be a result of abrasion against the second upper premolar tooth, especially if the tooth has a rostral enamel overgrowth. It is clear that the oral commissures and the bars of the mouth are susceptible to injury in sport horses and that further information is needed to determine the precise cause and nature of these injuries.

Due to the morphology of the cheek teeth, the mechanics of chewing, and the nature of the diet, horses tend to develop sharp enamel points along the buccal edge of the upper cheek teeth [5]. In addition, an enamel overgrowth (hook) is often present on the rostral edge of the upper second premolar [5]. It has been hypothesized that the lip commissures may be crushed between the bit and the rostral part of the second premolar and that the buccal mucosa can be compressed against the cheek teeth by a tight noseband, leading to abrasion by sharp enamel points. Buccal ulceration or evidence of previous ulceration adjacent to upper cheek teeth was reported in 94% of ridden Swedish horses [2].

The objectives of this study were to describe the prevalence and severity of oral lesions immediately before competition in a similar population of horses to those evaluated after competition in a previous study [2], and to investigate whether dental overgrowths were associated with the presence of ulcers and other lesions. First, we describe the presence and location of changes in pigmentation, scars, bruising, erosions, and ulcers in the lip commissures, buccal mucosa, and mandibular interdental spaces (bars) of the mouth of 342 dressage, show jumping, and eventing horses presented for an obligatory, pre-competition veterinary examination at a national competition in 2020; secondly, we seek associations between the presence and locations of specific oral lesions; and third, we seek associations between dental overgrowths and oral lesions.

## 2. Materials and Methods

### 2.1. Study Design

A population of 342 horses and ponies were evaluated during the Danish Equestrian Federation National Championship competitions in dressage, show jumping, and eventing between August and September 2020. An additional population of 82 ponies were evaluated during the Danish Equestrian Federation National Championship competitions in dressage for ponies in August 2021. The Veterinary Consultant for the Danish Equestrian Federation (DEF) conducted a visual intraoral examination of all horses and ponies prior to competition. The study protocol was approved by the board (Executive Committee) and administration (General Secretary and CEO of sport) of the DEF. The examiner (DEF Veterinary Consultant) and stewards on site were licensed by DEF who can request to examine a horse at any time. All riders accepted the examination for this study. All examinations were announced beforehand on the DEF event site; highlighted for the specific events. Onsite at the competitions, printed signs stated that data from examinations were being gathered for research purposes, and riders were made aware that their data were being recorded.

The following data were collected and recorded in an Excel sheet:

General information: date, discipline (dressage, show jumping, eventing), event.

Horse-specific information: type (horse/pony), identification information.

Pony-specific information: ponies and riders were classified according to the three DEF competition categories that are based on the pony’s height at the withers and the rider’s age:

- Category I: height 141–148 cm, rider ≤ 16 years, highest qualification requirements and level of competition.
- Category II: height 131–140 cm, rider ≤ 16 years, lower qualification requirements than Category I.
- Category III: height ≤ 130 cm, rider ≤ 13 years, lowest qualification requirements and level of competition.

For ponies in Category I the trainer of each pony/rider combination was recorded in 2020.

Overall, 342 horses are included in the study. The 27 horses in the Young Rider competition were the first “batch” to be examined during the 2020 season and only findings related to the commissures of the lips were recorded. After that, the protocol for examination was extended from only including the oral commissures to also include the buccal mucosa, bars, and dental arcades for the rest of the “batches” (315 horses).

Information related to the oral cavity: The presence of scarring/depigmentation, erosion/contusion, ulcers, and/or bleeding were recorded on the left and right sides of the skin (externally) and the mucosa (internally) at the lip commissures, the mucosa covering the bars, and the buccal mucosa. Recordings of the presence of scarring and/or depigmentation were combined with recordings of clearly demarcated circular or linear areas with lighter pigmentation than the natural color of the skin or mucosa [6], and the presence of healed wounds of variable depth. Fissures were recognized as deep scars that split the natural lining of the lips at the oral commissures. Ulcers were defined as lesions involving exposure of the dermis or underlying layers. Bleeding involved the presence of fresh blood in association with a lesion at the oral commissure. The presence of enamel overgrowths (hooks) on the rostral aspect of the upper second premolar teeth (#106 and/or #206) or sharp enamel points on the buccal aspect of the rostral one third of the left and right upper dental arcades were recorded.

Photographs: When ulcers were present, photographs were taken for subsequent analysis. MU stabilized the horse by placing a hand lightly over the nasal bone in the area between the nostrils and the eyes. The thumb from the other hand was placed inside the lower lip, lightly everting the lip laterally to expose the skin and mucosa of the oral commissure. An official/steward took the photo using a cell phone placed to avoid the shine/reflection from the mucosa.

Information from photographic analysis of ulcers from the 2020 population: Two evaluators, the Veterinary Consultant for the DEF (MU) and a veterinarian with a research specialty in wound pathology (LB), examined and scored the ulcers in a standardized manner according to the visual presentation of the tissue. The edge of the ulcer, defined as the ~2 mm margin, was categorized as normal, red erythematous, greyish, or thickened/unhealthy fibrous. The central bed of the ulcer was described as red/pink, pale red/grey/white, or dark red/brown/black. Additionally, fissures that interrupted the natural lining of the mouth to a depth of ≥0.2 cm were recorded as being representative of healed major wounds.

### 2.2. Statistical Analysis

Statistical associations between findings on the left and right sides of the oral cavity and associations between different lesion types were evaluated by simple chi-squared statistics (proc freq, SAS Institute). When cross-tabulations of factors and lesions contain combinations with less than five observations, *p*-values calculated from the chi-square distribution are inaccurate. Exact *p*-values were then calculated, using the exact option (proc freq, SAS Institute). The observational unit for evaluating different types of lesions was one horse, with the lesion considered as being present if it was found either unilaterally or bilaterally.

For the subset of ponies analyzed in 2020, the statistical association between ulcers and competition category was analyzed in the same way, using proc freq (SAS Institute). Exact *p*-values were calculated.

The effect of trainer for the subpopulation of Category I ponies was analyzed in a generalized, linear mixed model, using a logistic link and assuming a binomial distribution (proc glimmix, SAS Institute). Due to the relatively large number of different trainers, trainer was included in the model as a random effect. The *p*-value of the random effect was evaluated, using the cov-test-option in proc glimmix.

The effect of year for the ponies was evaluated by simple chi-square-statistics (proc freq, SAS Institute). No further analyses could be performed, since the difference between the results was so big for the two years.

## 3. Results

### 3.1. Relation between Findings on Left and Right Sides

For data collected in 2020, there were several types of lesions that, when present on one side of the mouth, had an increased risk of also being present on the other side (Table 1).

Additionally, if dental hooks or sharp enamel points were recorded on one side, they were likely to be present on the other side also (*p* < 0.0001) (Table 1).

### 3.2. Relation between Findings on the Skin and Mucosa of the Lip Commissures

At the lip commissures, if ulcers (*p* < 0.0001) and scarring/depigmentation (*p* < 0.0001) were present in the mucosa inside the mouth, there was an increased risk of finding similar lesions externally on the skin (Table 2).

### 3.3. Relation between Different Findings

Both cutaneous and mucosal ulcers were correlated to scarring/depigmentation (*p* < 0.0001).

Dental findings were significantly associated with the presence of scarring/depigmentation but were not associated with ulcers or erosion/contusion of the lip commissures. However, dental findings (hooks, enamel points) were associated with erosion/contusion of the buccal mucosa (*p* < 0.0001). Due to the low number of ulcers in this area, a *p*-value could not be calculated.

### 3.4. Relation between Findings at Different Locations

Erosion/contusion of the mucosa of the lip commissures and the bars were associated (*p* = 0.0002), and ulcers of the buccal mucosa and bars were also associated (*p* = 0.003).

### 3.5. Bleeding

No bleeding was recorded.

### 3.6. Pathology of Ulcers

Pathological classification of the 37 ulcers on the left and right sides indicated that for the ulcer edge there were 22 normal, 15 grey, and 0 red or white. For the ulcer bed the classification was 24 red, 8 pale, and 5 dark.

### 3.7. Relation between Size Category of Ponies and Trainer of a Pony/Rider Combination with Ulcers

For the 2020 subpopulation of 100 dressage ponies, the frequency of ulcers was significantly different between the three height categories (*p* = 0.007): 0% (0/24) for category III; 8.7% (2/23) for category II; and 26.4% (14/53) for category I.

A separate analysis of the 53 pony/rider combinations competing in Category I indicated they were trained by 27 trainers, with 1–5 pony/rider combinations per trainer. A significant effect of trainer on the occurrence of ulcers was shown (*p* = 0.03). For trainers with only one student, ulcers were found in 6/15 ponies (40%). For trainers with two students, ulcers were found in 1/10 ponies (10%). For trainers with 3 students, ulcers were found in 0/3 ponies (0%). Five trainers were each responsible for five pony/rider combinations, of which 7/25 ponies (28%) had ulcers. Ponies trained by two of these trainers had 0% oral ulcers. Overall, ponies trained by three trainers, each responsible for seven pony/rider combinations, accounted for half of the total cases.

### 3.8. Relation between Findings of Ulcers in Pony Dressage in 2020 and 2021

In the 2021 pony dressage championships, there were significantly fewer ponies with ulcers compared with 2020 (*p* = 0.0001). In 2020, 16/100 ponies in categories I, II, and III presented with ulcers (16%) compared with only 1/82 (1.2%) in 2021, which was a Category II pony.

For Category I ponies, 17/30 that competed in 2020 also competed in 2021 (57%) and 14 ponies competed with the same rider (82%) in both years. Three ponies that were excluded from competition due to ulcers in 2020 did not have ulcers in 2021.

## 4. Discussion

In 2020, the DEF inspected horses competing at the Danish National Championships for oral lesions in areas related to the position and action of the bit. Horses presenting with ulcers were not allowed to compete. This type of mandatory examination has serious consequences for competitors and, therefore, it is very important to use a standardized procedure for examining parameters related to equine welfare at competitions to ensure that the outcome is fair and reproducible. The reason for a mandatory examination was to promote equine welfare in horse sports by eliminating horses that presented with oral ulcers without further distinction of the cause of the ulcer. Prevention of lesions lies in the understanding of predictive markers and causative agents and, therefore, it is important to distinguish findings related to the use of the bit from other causes of similar lesions. The majority of lesions affecting areas related to the bit are believed to be associated with pressure or repeated microtrauma from the bit.

This study showed a significant correlation between findings of ulcers and scarring/depigmentation at the oral commissures, which serves as a clear indication that the two parameters are closely linked to each other and to the seat of the bit at the corners of the mouth. The reliability of changes in scarring/pigmentation has been investigated [6]. The findings indicated that there was wide variation in the natural oral pigmentation pattern, and it described the appearance of potentially pathological patterns of pigmentation. However, a few horses that had never worn a bit had pigmentation spots resembling those described as potentially pathological changes, which highlighted the difficulty of distinguishing whether pigment changes are related to bit use in individual horses. On the other hand, scarring was found to be a reliable indicator for previous problems with the use of the bit.

Scarring and depigmentation indicate previous problems with the bit, but evaluation in competition is concerned with welfare evaluation only on the day of competition. At population level, however, it is interesting to assess the frequency of lesions indicating past problems because they can serve as good indicators for the overall use of bits.

The presence of ulcers, scarring, depigmentation, or fissures on one side of the oral commissure was related to an increased risk of similar findings on the other side. The tendency for bilateral occurrence of the oral commissure lesions supports an exogenous causative agent, and in this anatomical location the bit is likely to be the culprit. The bilateral occurrence also suggests that, for many horse/rider combinations, problematic use of the bit is a general problem rather than being related to a left/right preference or handedness issue. Although erosion/contusion of the buccal mucosa was only observed in two horses, the tendency for a bilateral pattern was also seen for the buccal mucosa, but not the oral commissures.

The anatomical location of an ulcer is relevant when addressing causation, with differences being reported for those at the oral commissures, the bars, and the buccal mucosa. Lesions in and around the lip commissures have been described frequently and have been related to the use of snaffle bits, which is not surprising since this is the most commonly used bit in Europe [6]. Jointed snaffles act primarily on the commissures and the tongue. Tight nosebands have also been associated with commissural lesions perhaps related to holding the bit up into the commissures [6]. Lesions of the bars have been related to the use of a curb bit with a port that circumvented the effect of the tongue in protecting the bars or an unjointed snaffle [7].

Thoroughbred racehorses gallop with the head and neck in an extended position to straighten the airway and facilitate breathing. The bit, which is usually a jointed snaffle, is pulled up into the commissures where it can exert considerable pressure, resulting in a high frequency of severe commissural lesions in racehorses [4]. These authors suggested that the commissures may be crushed between the bit and the first upper-premolar tooth, especially if the tooth has a rostral enamel overgrowth. The data presented here did not show a link between dental findings, such as enamel overgrowths, with ulcers or erosion/contusion at any location, which argues against crushing of the lips against the premolar teeth being responsible for commissural ulcers. For a full understanding and evaluation of the relation between lesions and dental findings, more research is needed. Tell et al. [2] found a tendency for the frequency of commissural ulcers to be higher in ridden versus unridden horses. Other studies indicated that ulcers occurred primarily in horses competing at a high level in the disciplines of dressage, eventing, Icelandic competitions [3], trotting races [8], galloping races [4], and polo [4]. These findings are supported by our findings in the subpopulation of dressage ponies, where the frequency of ulcers was 26.4% for Category I, which is the highest level, 8.7% for category II, and 0% for category III.

Badly fitted bits and poor riding have been blamed for ulceration. Therefore, it is relevant to evaluate how the rider interacts with the horse through the bit, which is a reflection of the rider’s skill and training methodology. The orientation of the rein tension vector depends on a number of factors, including the type of bit, position and angulation of the horse’s head and neck, and the position and movements of the rider’s hands. Gag bits allow the mouthpiece to slide up the cheekpiece so that rein tension pulls the mouthpiece higher and stretches the lip commissures while reducing pressure on the tongue and bars. In polo ponies, which are usually ridden in gag bits, 85% of oral lesions have been reported to affect the commissures [4]. For horses competing in sports such as dressage, show-jumping, and eventing, the head and neck position in relation to the rider’s hand are determinants of whether rein pressure is distributed more to the commissures or to the tongue and bars.

In our study, all recorded ulcers were classified as chronic, non-bleeding wounds. It is important to understand that ulcers affecting areas related to the bit often develop over a substantial period of time as a result of direct pressure or repetitive microtraumas between the bit and the oral tissue rather than being caused by a sudden acute tearing of tissue, although this cannot be ruled out as a cause of the subsequent development of a chronic wound. Minor acute tears or disruptions of the bed of a chronic wound can result in the ulcer persisting over time, and ongoing irritation can cause inflammation, recognized as redness.

Blood coming from the horse’s mouth takes on special importance under International Equestrian Federation rules that, in most cases, require elimination of a horse that has blood in its mouth but with some differences between disciplines. Chronic non-bleeding ulcers from use of the bit are not specifically addressed in the regulations. The absence of bleeding in the horses in this study, even in those with severe lesions, implies that it may be insufficient for equestrian sport governing bodies to only address bleeding as an evaluation factor. Abrasion of the skin should equally be seen as a decisive factor in an evaluation of fitness to compete.

The trainer instructs the rider and corrects their posture, movements, and use of the aids which affect the body position of the horse. The data in this study showed that, for the trainers with the largest number of students riding ponies at the event, the frequency of ulcers ranged from 0% to 80% and a small subset of trainers within this group trained 50% of all ponies with ulcers. However, many factors may influence the significance of the trainer’s role, for example, how long the trainer has been responsible for the pony/rider combination, whether more than one trainer is used by the rider, and the type of bit used during daily training. Further studies are needed to investigate the trainer’s influence on the occurrence of bit-related ulcers. In the subset of ponies evaluated in successive years, there was a highly significant reduction in the number of ponies with ulcers, which suggests that awareness about the problem caused riders to focus on methods to greatly reduce the occurrence of ulcers. Active monitoring is recommended to encourage all responsible persons to take steps to improve equine welfare.

Little is known about iatrogenic causes of oral injury or allergies to specific metals in the alloys used in bit manufacture. Human orthodontic data indicate that both nickel and titanium can be allergenic [9]. Nickel allergy is a contact dermatitis that causes a delayed hypersensitivity immune response with signs of itching, redness, rash, dry patches, and tissue swelling. Nickel constitutes 70–90% of stainless steel. Allergies to titanium or its alloys are uncommon in people, but an existing allergy to other metals is a predisposing factor [10]. Allergic reactions to metals are unlikely to be a frequent cause of equine oral lesions but should not be completely discounted due to the close contact of the bit with the lips and oral commissures. It is possible that some factors contributing to oral lesions have not yet been identified.

A limitation of this study is the small number of observations in some groups.

## 5. Conclusions

In this study, oral lesions were recorded in 342 horses and ponies presented for pre-competition veterinary examination at a national competition. Oral lesions were primarily located inside the lip commissures without associated bleeding. Lesions thought to be related to bit use occurred more frequently at higher levels of competition in a sub-population of dressage ponies. The presence of lesions on one side of the oral commissure was related to an increased risk of similar findings on the other side, and the anatomical location suggests bit-related trauma. The lesions were not associated with enamel overgrowth. The findings support the need for a consistent and reproducible protocol to evaluate riding horses for lesions in the bit area of the lip commissures to be used by sport governing bodies to determine whether individual horses are fit to compete, and it suggests that greater awareness of the problem might influence the prevalence of oral ulcers.

## Figures and Tables

**Table 1:** Oral lesions recorded in 342 horses and ponies evaluated prior to competition. *p* < 0.05 indicates that if there is a lesion on one side, then there is an increased risk of a lesion on the other side.

Lesion	No Lesion (%)	Lesion 1 Side (%)	Lesion 2 Sides (%)	*p*-Value
Ulcer inside commissure	314 (92%)	20 (6%)	8 (2%)	<0.0001
Ulcer outside commissure	326 (95%)	13 (4%)	3 (1%)	<0.0001
Erosion/contusion inside commissure	320 (94%)	21 (6%)	1 (0.3%)	0.22
Erosion/contusion outside commissure	341	1	0	
Scarring/depigmentation inside commissure	240 (70%)	71 (21%)	31 (9%)	<0.0001
Scarring/depigmentation outside commissure	273 (80%)	52 (15%)	17 (5%)	<0.0001
Ulcer buccal mucosa *	314	1	0	-
Erosion/contusion buccal mucosa *	313 (99.4%)	0	2 (0.6%)	<0.0001
Scarring/depigmentation buccal mucosa *	315	0	0	-
Ulcer on bar *	314	1	0	-
Erosion/contusion on bar *	309 (98%)	6 (2%)	0	1
Scar on bar *	315	0	0	-
Dental arcade sharp enamel points	269 (85%)	6 (2%)	40 (13%)	<0.0001
Dental arcade hooks 2nd premolar	275 (87%)	8 (3%)	38 (10%)	<0.0001

* only 315 observations because lesions were not recorded at one event.

**Table 2:** Associations between lesions of different types and in different locations.

Lesion 1	Lesion 2	Neg–Neg	Neg–Pos	Pos–Neg	Pos–Pos	*p*-Value
Ulcer inside commissure	Ulcer outside commissure	313	1	13	15	<0.0001
Ulcer inside commissure	Scar/depigmentation inside commissure	240	74	0	28	<0.0001
Ulcer inside commissure	Scar/depigmentation outside commission	262	52	11	27	<0.0001
Ulcer outside commissure	Scar/depigmentation inside commissure	240	86	0	16	<0.0001
Ulcer outside commissure	Scar/depigmentation outside commission	273	53	0	16	<0.0001
Erosion/contusion inside commissure	Erosion/contusion bar	291	2	18	4	0.0002
Scar/depigmentation inside commissure	Scar/depigmentation outside commission	233	7	40	62	<0.0001
Ulcer buccal mucosa	Ulcer bar	314	0	0	1	0.003

*p* < 0.05 indicates a correlation between the combinations of types and location of lesions in the table. Neg: negative; Pos: positive.

## Data Availability

Full data available from the first author.
